# Norepinephrine potentiates the efficacy of volume expansion on mean systemic pressure in septic shock

**DOI:** 10.1186/s13054-021-03711-5

**Published:** 2021-08-21

**Authors:** Imane Adda, Christopher Lai, Jean-Louis Teboul, Laurent Guerin, Francesco Gavelli, Xavier Monnet

**Affiliations:** AP-HP, Service de médecine intensive-réanimation, Hôpital de Bicêtre, DMU CORREVE, Inserm UMR S_999, FHU SEPSIS, Groupe de Recherche Clinique CARMAS, Université Paris-Saclay, 78, Rue du Général Leclerc, 94270 Le Kremlin-Bicêtre, France

**Keywords:** Vasopressors, Heart–lung interactions, Fluid balance, Cardiac preload, Venous return

## Abstract

**Background:**

Through venous contraction, norepinephrine (NE) increases stressed blood volume and mean systemic pressure (Pms) and exerts a “fluid-like” effect. When both fluid and NE are administered, Pms may not only result from the sum of the effects of both drugs. Indeed, norepinephrine may enhance the effects of volume expansion: because fluid dilutes into a more constricted, smaller, venous network, fluid may increase Pms to a larger extent at a higher than at a lower dose of NE. We tested this hypothesis, by mimicking the effects of fluid by passive leg raising (PLR).

**Methods:**

In 30 septic shock patients, norepinephrine was decreased to reach a predefined target of mean arterial pressure (65–70 mmHg by default, 80–85 mmHg in previously hypertensive patients). We measured the PLR-induced increase in Pms (heart–lung interactions method) under high and low doses of norepinephrine. Preload responsiveness was defined by a PLR-induced increase in cardiac index ≥ 10%.

**Results:**

Norepinephrine was decreased from 0.32 [0.18–0.62] to 0.26 [0.13–0.50] µg/kg/min (*p* < 0.0001). This significantly decreased the mean arterial pressure by 10 [7–20]% and Pms by 9 [4–19]%. The increase in Pms (∆Pms) induced by PLR was 13 [9–19]% at the higher dose of norepinephrine and 11 [6–16]% at the lower dose (*p* < 0.0001). Pms reached during PLR at the high dose of NE was higher than expected by the sum of Pms at baseline at low dose, ∆Pms induced by changing the norepinephrine dose and ∆Pms induced by PLR at low dose of NE (35.6 [11.2] mmHg vs. 33.6 [10.9] mmHg, respectively, *p* < 0.01). The number of preload responders was 8 (27%) at the high dose of NE and 15 (50%) at the low dose.

**Conclusions:**

Norepinephrine enhances the Pms increase induced by PLR. These results suggest that a bolus of fluid of the same volume has a greater haemodynamic effect at a high dose than at a low dose of norepinephrine during septic shock.

**Supplementary Information:**

The online version contains supplementary material available at 10.1186/s13054-021-03711-5.

## Take-home message

Norepinephrine enhances the increase in mean systemic pressure induced by a passive leg raising. This suggests that norepinephrine and fluid therapy may exert cumulative haemodynamic effects in septic shock patients.

## Background

Volume expansion and norepinephrine (NE) are the main pillars of the haemodynamic support in septic shock, and their cardiovascular properties have been extensively studied. In particular, some studies have focused on the NE effects on venous return [1–5]. They have shown that by constricting veins as it constricts arteries, NE reduces the venous capacitance and increases the mean systemic filling pressure (Pms) [1–3]. The resulting increase in cardiac preload is of significant amplitude in clinical practice [4, 5], so that NE increases cardiac output in case of preload responsiveness [2, 5].

In addition to this fluid-like effect, NE might also potentiate the effects of volume expansion on Pms in a “cumulative” way. By reducing the capacitance of the venous tank, NE might enhance the increase in stressed blood volume provoked by fluid administration [3]. The same volume of fluid might increase Pms to a larger extent at a higher than at a lower dose of NE. In such a case, administering both NE and fluid would exert an effect on Pms which would be larger than the addition of the effect of each treatment. NE would potentiate the effects of volume expansion on Pms.

The present study investigated this hypothesis. Its purpose was to test, in patients with septic shock, if the amplitude of the change in Pms induced by a volume challenge would be larger at a higher than at a lower dose of NE, suggesting cumulative effects between NE and volume expansion. For this purpose, we used the estimation of Pms and resistance to venous return developed by Maas et al. [1]. The volume challenge was performed by passive leg raising (PLR), which mimics volume expansion by exerting comparable effects on venous return determinants [6] and which is fully reversible [7].

## Methods

### Study population

This study was conducted in the 25-bed medical intensive care unit of Paris-Saclay university, Bicêtre hospital, from March to September 2018. It was approved by the institutional review board of our institution (Comité pour la protection des personnes Ile-de-France VII). Patients or their next of kin were informed about the study and accepted to participate.

Patients were included if they met all the following criteria: septic shock [7], continuous intravenous administration of NE, mechanical ventilation in the volume assist-control mode (Evita 4 or V500, Dräger, Lübeck, Germany), haemodynamic monitoring by a PiCCO2 device (PULSION Medical Systems, Feldkirchen, Germany), haemodynamic stability as defined by no change in the mean arterial pressure and in cardiac index (CI) > 10% for at least 30 min [2], decision of the attending physicians to decrease the dose of NE in order to reach a predefined target of mean arterial pressure (65–70 mmHg by default, 80–85 mmHg in previously hypertensive patients [8]). Patients were not included consecutively but depending on the availability of the investigators.

Patients were excluded in case of age < 18 years, pregnancy, head trauma (contraindication to PLR) and intra-abdominal hypertension [9] or venous compression stockings (both responsible for some false-negatives of the PLR test).

### Study design

**Fig. 1:**
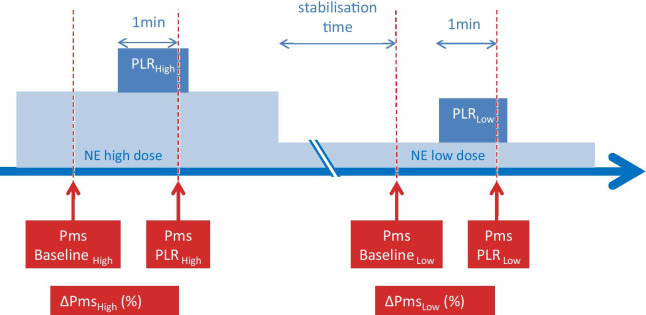
Study design. Baseline_Low_: baseline at the low dose of norepinephrine, Baseline_High_: baseline at the high dose of norepinephrine, NE: norepinephrine, PLR_Low_: passive leg raising at the low dose of norepinephrine; PLR_High_: passive leg raising at the high dose of norepinephrine, Pms: mean systemic pressure, ∆Pms: changes in mean systemic pressure induced by passive leg raising

At baseline, we performed a first set of measurements, including Pms and resistance to venous return as shown in Fig. 1. A PLR was then performed by moving the patient from the semi-recumbent position at 45° to a position where the legs are elevated at 45° and the trunk is horizontal [10]. After one minute and stabilisation of CI, transpulmonary thermodilution was performed. Then, a second set of measurements was performed as at baseline, including Pms and resistance to venous return. The patient was then moved back to the initial semi-recumbent position.

After the first set of measurement, the dose of NE was decreased in one step, as decided by the clinician in charge of the patient, with the aim of reaching a mean arterial pressure corresponding to the prescribed target [11]. If this decrease induced hypotension, the dose of NE was increased. In any case, after the last change of the dose of NE, stability was allowed for at least 30 min. Once the dose of NE was decreased, it was kept stable for the rest of the study.

After stabilisation, a transpulmonary thermodilution was performed, and a series of measurements was performed as at the high dose of NE. A second PLR was started. One minute later, measurements were recorded again (Fig. 1). The patient was returned to the semi-recumbent position. All other treatments were kept unchanged during the study period. The same operator performed all the measurements of the patient in order to avoid an operator-dependent effect [12].

### Measurements

#### Haemodynamic measurements

The arterial and central venous pressure sensors were placed at the level of the mid-axillary line, and zeroing was performed against atmospheric pressure. Airway pressure was measured at the proximal extremity of the endotracheal tube. Arterial pressure, central venous pressure (CVP), and airway pressure were continuously computerised using the HEM 4.2 data acquisition software (Notocord, Croissy sur Seine, France). CI was measured by the PiCCO2 device through transpulmonary thermodilution [13] and pulse contour analysis [14]. The beat-to-beat values of stroke volume derived from pulse contour analysis performed by the PiCCO2 device were computerised by using the PiCCOWin 4.0 software (Pulsion Medical Systems, Feldkirchen, Germany). Calibration of pulse contour analysis-derived stroke volume was performed by using transpulmonary thermodilution, with injection of three 15-mL cold saline boluses [15, 16]. Through transpulmonary thermodilution, we also measured the cardiac function index (estimation of the left ventricular systolic function) [17].

#### Pms and resistance to venous return

Pms and the resistance to venous return were determined by constructing an estimated venous return curve by using the haemodynamic effects of heart–lung interactions. This “heart–lung interactions method” has been previously described [1, 2].

Briefly, it is based on the principle that the venous return curve is the relationship between right atrial pressure and venous return according to the Guyton’s model. A series of two 15-s end-inspiratory occlusions and of two 15-s end-expiratory occlusions were performed at the level of positive end-expiratory pressure (PEEP) which was adjusted before the beginning of the study for reaching a plateau pressure of 28–30 cmH_2_O and kept constant throughout the entire study procedure.

The four occlusions were performed in a random order. During respiratory occlusions, the extreme value of CI (minimal for inspiratory occlusions, maximal for expiratory occlusions) measured by pulse contour analysis reached was averaged over the two last seconds of occlusions. The extreme value of CVP (minimal for expiratory occlusions, maximal for inspiratory occlusions) was recorded at the same time. Each ventilatory occlusion allowed us to obtain a couple of measurements of CI and CVP.

The couple of CI and CVP values was reported on a graph (Excel, Microsoft, Redmond, WA) with CVP on the x-axis (as an estimate of right atrial pressure) and CI on the y-axis (as an estimate of venous return, since venous return and cardiac output are equal at steady state) (Additional file 1: figure S1). Then, the regression line between these points was computed by using the least-squares method (Excel, Microsoft, Redmond, WA). Pms was estimated as the pressure at the x-intercept of the regression line, as shown in Additional file 1: figure S1. The resistance to venous return was estimated from the inverse of the slope of the regression line.

### Data analysis

In order to test our hypothesis that NE potentiates the effects of volume expansion on Pms, we compared for each patient the value of Pms that was actually observed during PLR_High_ with the value that would be expected during PLR_High_ if it resulted only from the sum of the effects of PLR and of increasing the dose of NE (sum of Pms at Baseline_Low_ + change in Pms induced by changing the dose of NE + change in Pms induced by PLR_Low_). If both values were significantly different, we considered that our hypothesis was valid.

In order to test the same hypothesis, we also compared in each patient the relative changes in Pms induced by PLR (ΔPms) at the high (∆Pms_High_) and the low dose (∆Pms_Low_) of NE. If ∆Pms_Low_ was lower than ∆Pms_High_, we considered that decreasing NE reduced the efficacy of a volume challenge on Pms.

The indexed arterial resistance was calculated as (mean arterial pressure – Pms)/CI and the indexed venous resistance as (Pms − CVP)/CI. Preload responsiveness at the highest and the lowest dose of NE was defined by an increase in CI ≥ 10% during PLR_High_ and PLR_Low_, respectively [10].

### Statistical analysis

Taking into account an α risk of 5% and a β risk of 20%, considering that the change in ΔPms induced by the modification in NE would be 8 ± 15% [5, 6], it was planned to include 30 patients. The normality of data distribution was assessed visually.

Data are expressed as median [interquartile range], mean [standard deviation] or frequency (n, %), as appropriate. ∆Pms_High_ and ∆Pms_Low_ were compared using a Wilcoxon test. For the other variables, the comparisons between the different times of the study were performed using a Friedman test or ANOVA for repeated measurements, followed by a pairwise comparison of variables according to Conover [18] as appropriate. Comparisons between subgroups were performed using a Mann–Whitney *U* test. The relationship between variables was tested using the Spearman correlation. There were no missing data. The statistical analysis was performed using MedCalc 11.0.0 software (MedCalc, Mariakerke, Belgium) and reviewed by statisticians of our institution.

## Results

### Patient characteristics

Thirty patients were included 6.0 [4.4] days after the onset of septic shock. No patient met the exclusion criteria. No patients received a catecholamine other than NE (Table 1). Twelve (40%) patients were known to have hypertension. All patients received sedation, and 23 (77%) received neuromuscular blocking agents. For five (17%) patients, continuous veno-venous haemofiltration was in progress during the measurements, without fluid removal. Seven (23%) patients were in atrial fibrillation. Fifteen (50%) patients received corticosteroids (hydrocortisone 200 mg/day) (Table 1). Considering all timepoints in the study, the coefficient of determination of regression lines built for estimating Pms was *r*^2^ = 0.91 [0.83–0.98].

**Table 1:** Patient characteristics at baseline

Age (years)	70 [12]
M/F ratio	25/5
SAPS II	55 [14]
Source of sepsis	
Lung (*n* (%))	22 (73%)
Abdomen (*n* (%))	6 (20%)
Catheter (*n* (%))	2 (7%)
Lactate (mmol/L)	2.6 [3.8]
PaO_2_/FiO_2_ (mmHg)	142 [132–198]
Tidal volume (mL/kg predicted body weight)	4.8 [0.9]
Respiratory rate (breaths/min)	30 [16]
PEEP (cmH_2_O)	11 [4]
Plateau pressure (cmH_2_O)	26 [4]
Compliance of the respiratory system (mL/cmH_2_O)	36 [10]

*N* = 30. Data are expressed as numbers (%) or mean [standard deviation] or median [interquartile range]

F_i_O_2_, inspired fraction of oxygen; M/F, males/females; P_a_O_2_, arterial oxygen partial pressure; PEEP, positive end-expiratory pressure; SAPS: Simplified Acute Physiologic Score

### Effects of PLR_High_

**Fig. 2:**
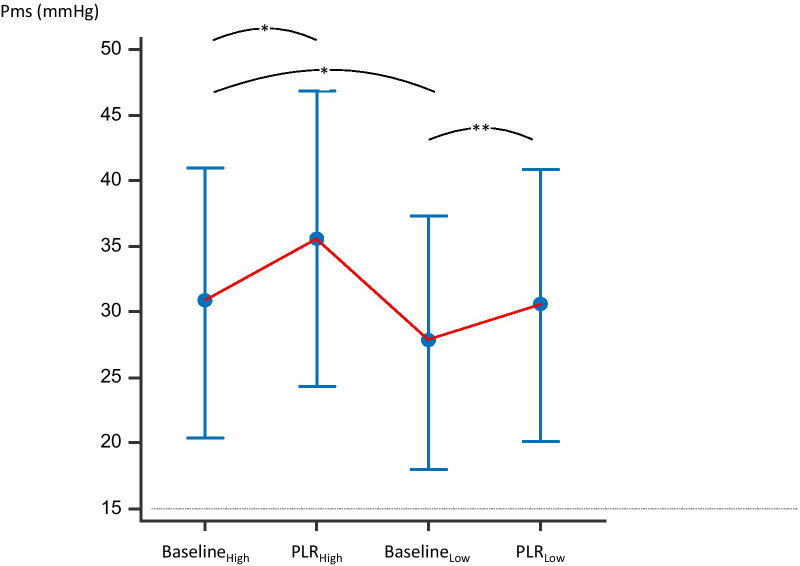
Changes in mean systemic pressure during the study protocol. Baseline_Low_: baseline at the low dose of norepinephrine, Baseline_High_: baseline at the high dose of norepinephrine, PLR_Low_: passive leg raising at the low dose of norepinephrine; PLR_High_: passive leg raising at the high dose of norepinephrine, Pms: mean systemic pressure. *p < 0.05 vs. Baseline_High_, **p < 0.05 vs. Baseline_Low_. N = 30, mean [standard deviation]

The first PLR, performed at the highest dose of NE, significantly increased Pms by 13 [9–19]% compared to Baseline_High_ (Table 2, Fig. 2). The resistance to venous return did not significantly change. During PLR_High_, CI significantly increased by 6 [2–10]% and it increased by ≥ 10% in 8 (27%) patients, indicating preload responsiveness (Table 2).

**Table 2:** Haemodynamic variables during the study protocol

	High dose of NE		Low dose of NE	
	Baseline	PLR	Baseline	PLR
SAP (mmHg)	141 [25]	146 [25]	120 [17] ^b^	127 [23]^c^
DAP (mmHg)	60 [9]	63 [11]^a^	54 [7]^b^	58 [8]^c^
MAP (mmHg)	88 [12]	94 [13]	76 [8]^b^	80 [10]^c^
Heart rate (beats/min)	93 [16]	96 [15]	92 [14]	96 [14]
CVP (mmHg)	13.0 [5.0]	15.8 [5.8]^a^	12.4 [5.2]	14.3 [5.9]^c^
Pms (mmHg)	30.7 [10.3]	35.6 [11.2]^a^	27.7 [9.7]^b^	30.5 [10.4]^c^
Pms-PVC (mmHg)	17.6 [9.8]	19.7 [11.2]	15.3 [9.7]^b^	16.2 [9.8]
Indexed venous resistance (mmHg.min.m^2^/L)	5.4 [2.7]	5.5 [2.9]	4.9 [2.7]	4.7 [2.3]
Indexed arterial resistance (mmHg.min.m^2^/L)	17.7 [8.3]	16.4 [10.0]^a^	15.6 [6.1]^b^	14.4 [6.1]
Cardiac index (L/min/m^2^)	3.24 [0.9]	3.57 [0.9]^a^	3.08 [0.7]^b^	3.43 [0.8]^c^
Dose of NE (μg/kg/min)	0.32 [0.2–0.6]	0.32 [0.2–0.6]	0.26 [0.1–0.5]^b^	0.26 [0.1–0.5]
Cardiac function index (/min)	4.8 [1.3]		4.9 [1.5]	

*N* = 30. Data are expressed as mean [standard deviation]

CVP, central venous pressure; DAP, diastolic arterial pressure; MAP, mean arterial pressure; NE, norepinephrine, Pms, mean systemic pressure; SAP, systolic arterial pressure

^a^*p* < 0.05 PLR at high dose *vs*. baseline at high dose

^b^*p* < 0.05 baseline at low dose *vs*. baseline at high dose

^c^*p* < 0.05 baseline at low dose *vs*. PLR at low dose

### Effects of decreasing NE

The dose of NE decreased from 0.32 [0.18–0.62] to 0.26 [0.13–0.50] µg/kg/min (*p* < 0.001) (Table 2). Additional file 1: table S1 describes the NE change per patient during the study. The time between the decrease in NE and Baseline_Low_ was 38 [32–48] min. During this time, the fluid balance was − 1 [− 18 to 22] mL.

With this modification of NE, the mean arterial pressure decreased by 10 [7–20]% (*p* < 0.001). Pms decreased by 9 [4–19]% (*p* < 0.001) (Table 2). CI decreased by 11 [3–16]% (*p* < 0.001).

The NE-induced change in Pms (expressed either in absolute value or percentage) was significantly correlated with the amplitude of the change in NE dose (*r* = 0.66, *p* < 0.01). It was correlated with the diastolic arterial pressure at Baseline_High_ (*r* = 0.62, *p* = 0.0003) and with the Pms at Baseline_High_ (*r* = 0.48, *p* < 0.01).

### Effects of PLR_Low_

PLR_Low_ significantly increased Pms by 11 [6–16]% compared to Baseline_Low_ (Table 2, Fig. 1). The resistance to venous return did not change significantly. During PLR_Low_, CI significantly increased by 10 [7–15]% on average and it increased by ≥ 10% in 15 (50%) patients (Table 2).

### Comparison between ΔPms induced by PLR_Low_ and PLR_High_

**Fig. 3:**
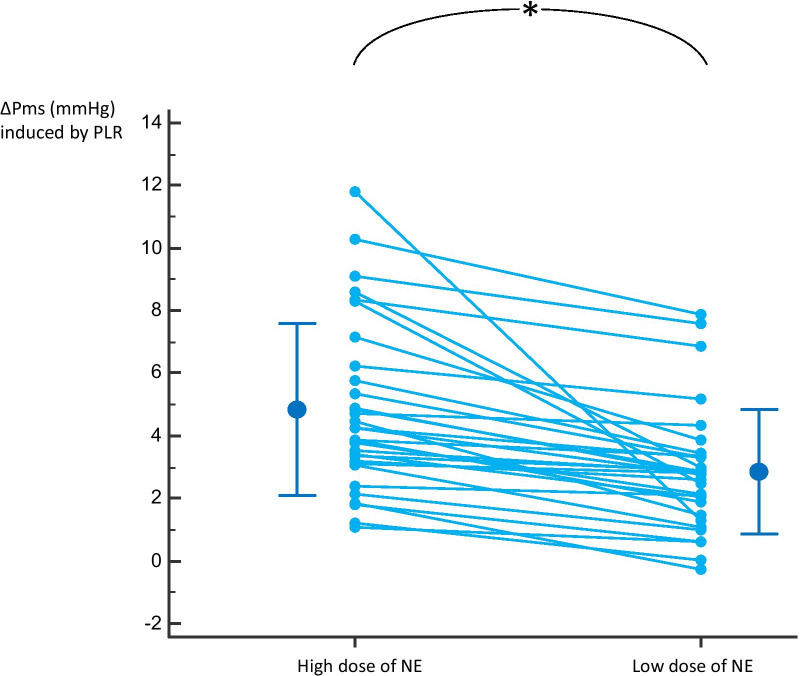
Changes in mean systemic pressure (∆Pms) induced by passive leg raising (PLR) at the highest and the lowest dose of norepinephrine (NE). *N* = 30, individual values and mean [standard deviation]

Pms at PLR_High_ was significantly higher than the sum: Pms at Baseline_Low_ + (Pms at Baseline_High_ − Pms at Baseline_Low_) + (Pms at PLR_Low_ − Pms at Baseline_Low_) (35.6 [11.2] mmHg vs. 33.6 [10.9] mmHg, respectively, *p* < 0.01). The ΔPms induced by PLR_High_ was significantly larger than the ΔPms induced by PLR_Low_ (13 [9–18] % *vs*. 11 [6–15]%, *p* < 0.001) (Fig. 3). The increase in CI induced by PLR_Low_ was significantly larger than the increase in CI induced by PLR_High_ (*p* < 0.01) (Table 2).

ΔPms_High_ − ΔPms_Low_ was not significantly correlated with the NE-induced ∆Pms (*p* = 0.30), with the change of the NE dose from Baseline_High_ to Baseline_Low_ (*p* = 0.23), with the diastolic arterial pressure at Baseline_High_ (*p* = 0.88), with the NE-induced change in mean arterial pressure (*p* = 0.56), with the PEEP level at baseline (*p* = 0.68), and with the airway driving pressure (*p* = 0.39). It was correlated with the airway plateau pressure at baseline (*r* = 0.45, *p* = 0.02). ΔPms_High_ − ΔPms_Low_ was not different between the half of the population with the smallest drop in norepinephrine and the half with the greatest drop (1.15 [0.52–1.51] mmHg and 2.07 [1.16–2.41] mmHg, respectively, *p* = 0.06).

The change of Pms from Baseline_High_ to Baseline_Low_ was significantly correlated with the simultaneous change in the NE dose (*r*^2^ = 0.34, *p* < 0.01) (Additional file 1: figure S2).

## Discussion

This study suggests that the PLR-induced increase in Pms (∆Pms) is larger at a higher dose of NE compared to a lower dose. The ∆Pms induced by PLR at a higher dose of NE was larger than the addition of the effects on Pms of changing the dose of NE and performing a PLR at a lower NE dose. It also confirms that decreasing the dose of NE significantly decreases Pms (“fluid-like” effect of NE). These results suggest that NE improves the haemodynamic efficacy of fluid administration.

NE is the first-line vasopressor in septic shock [19]. While NE is mainly used for restoring arterial pressure through arterial vasoconstriction, its effects on the venous circulation are likely important. The stimulation of the venous α-receptors induces venoconstriction, which increases the stressed blood volume and reduces the unstressed blood volume. Datta and Magder have evidenced this for the first time in animals [3]. More recently, some methods were developed for investigating the determinants of venous return in critically ill patients. In particular, Maas and co-workers elegantly described the heart–lung interactions method that we used in the present study [1, 20]. These studies conducted in septic shock patients confirmed that NE increases Pms [1, 2]. It also increases the resistance to venous return but to a smaller extent. As a result, NE increases the venous return and cardiac output in preload-dependent patients [2, 5]. Our study confirms these previous findings; Pms was significantly higher at the highest dose of NE. At this dose, the proportion of preload responsive cases was lower, and the PLR-induced increase in CI was also smaller. In this regard, the effects of NE on venous return mimic those of fluid administration.

The present study extends these findings. In septic shock patients, the increase in Pms induced by PLR, which mimics fluid administration, was significantly larger at the highest dose of NE than at the lowest. This suggests that at the highest dose of NE, the PLR increased the proportion of stressed blood volume to a larger extent than at the lowest dose. PLR (or fluid administration) at the lowest degree of vein constriction still increases the proportion of stressed blood volume, but to a lower extent than the same volume at high dose. In other words, the blood transfer of PLR might occur on a constricted venous network, which enhanced its haemodynamic efficacy. This corroborates the observation made by Harrois et al. that NE decreases fluid requirements during fluid resuscitation of uncontrolled haemorrhagic shock in mice [21]. The present study reports such an effect for the first time in septic shock patients despite the decrease in the dose of NE was small. Of note, the PLR-induced change in Pms was not correlated with the amplitude of the dose decrease in NE. This reflects the fact that the vascular reactivity is very variable from one patient to another, even at this post-acute phase of septic shock.

The PLR was used in order to reproduce the haemodynamic effects of fluid administration but in a reversible way. Otherwise, the comparison of the haemodynamic condition at the two NE doses would not have been possible. In fact, the volume of venous blood transferred towards the heart chambers during PLR might have been different under the two doses of NE. Indeed, at the highest dose, the volume of the constricted splanchnic and inferior limbs venous compartment was less, such that less blood volume might have been mobilised during PLR than at the lowest dose. Nevertheless, if it was the case, this would tend to reduce (not increase) the PLR-induced increase in Pms, which even reinforces the conclusions of the study.

In the intensive care unit, a positive fluid balance is deleterious and increases mortality [22–24]. The present results suggest that a possible interest of NE would be to limit the volume of fluid administered [21]. Together with the fact that it can restore arterial pressure rapidly in case of life-threatening hypotension, this may support the strategy to infuse NE early in the course of septic shock [25]. Septic shock resuscitation in the first hours requires fluid administration in order to correct hypoperfusion. Co-administration of fluid and NE by the effect described in our study, might increase the fluid efficacy on venous return and correct hypoperfusion faster in septic shock patients. This is in concordance with the observation that an early administration of NE reduced the total cumulative fluid balance and was associated with a reduction of mortality [24, 26].

Besides the methodological bias discussed above, our study has limitations. First, we investigated decreases rather than increases of NE in patients that had been already resuscitated. This was justified by the need of performing measurements during haemodynamic stability, which would have been impossible to observe in hypotensive patients at the initial phase of septic shock. Even though there is no pharmacological reason why the venous effects of NE should differ in direction when its dose is increased rather than decreased, performing our study in patients who would need an increase in the dose of NE, i.e. hypotensive patients, may have led to larger dose changes. Second, the change in NE dose was decided by the attending physicians in order to target their predefined goal of a mean arterial pressure. The dose decrease was not standardised, as it would have been possible in animal studies. Third, NE might exert a slight inotropic effect [4], which would reduce Pms and counterbalance the increase in Pms at the highest NE dose. The lack of change in the cardiac function index belies this assumption. However, it cannot be ruled out that left ventricular contractility may have increased anyway, because the cardiac function index, like the left ventricular ejection fraction [27], tends to decrease as ventricular afterload increases.

## Conclusions

This study showed that NE enhances the increase in Pms induced by a PLR manoeuvre. These results suggest that a bolus of fluid of the same volume has a greater haemodynamic effect at a high dose than at a low dose of norepinephrine during septic shock.

## Supplementary Information

**Additional file 1: figure S1**: Heart–lung interactions method for estimating the determinants of venous return. Example of the estimation of the venous return curve and its determinants with the heart–lung interactions method in a typical patient. **figure S2**: Correlation between the changes in mean systemic pressure induced by decreasing the dose of norepinephrine and the amplitude of the dose decrease. The change in the mean systemic pressure is on the y axis and the amplitude of the dose decrease on the x axis. **table S1**: Norepinephrine change per patient. This table describes the dose of NE before and after its decrease.

## Data Availability

The datasets used during the current study are available from the corresponding author on reasonable request.

## Abbreviations

CI: Cardiac index
CVP: Central venous pressure
ΔPms_High_: PLR-induced increase in Pms at the highest dose of NE
ΔPms_Low_: PLR-induced increase in Pms at the lowest dose of NE
NE: Norepinephrine
PEEP: Positive end-expiratory pressure
PLR: Passive leg raising
Pms: Mean systemic pressure
