# Supplementary material for: Norepinephrine potentiates the efficacy of volume expansion on mean systemic pressure in septic shock
Source: Crit Care. 2021 Aug 21;25:302. doi: 10.1186/s13054-021-03711-5 (PMC8379760; doi:10.1186/s13054-021-03711-5)
Supplement: Supplementary file 1 — Additional file 1: figure S1: Heart–lung interactions method for estimating the determinants of venous return. Example of the estimation of the venous return curve and its determinants with the heart–lung interactions method in a typical patient. figure S2: Correlation between the changes in mean systemic pressure induced by decreasing the dose of norepinephrine and the amplitude of the dose decrease. The change in the mean systemic pressure is on the y axis and the amplitude of the dose decrease on the x axis. table S1: Norepinephrine change per patient. This table describes the dose of NE before and after its decrease. [file 13054_2021_3711_MOESM1_ESM.doc]

Norepinephrine potentiates the efficacy of volume expansion on mean systemic pressure in septic shock

Imane ADDA^1^, Christopher LAI^1^, Jean-Louis TEBOUL^1^, Laurent GUERIN^1^, Francesco GAVELLI^1^, Xavier MONNET^1^

^1^Université Paris-Saclay, AP-HP, Service de médecine intensive-réanimation, Hôpital de Bicêtre, DMU CORREVE, Inserm UMR S_999, FHU SEPSIS, Groupe de recherche clinique CARMAS, Le Kremlin-Bicêtre, France

Supplementary file

Supplementary table 1: Norepinephrine change per patient during the study

| Patient # | | | Dose of NE at Baseline_High_ (µg/kg/min) | Dose of NE at Baseline_Low_ (µg/kg/min) |
| --- | --- | --- | --- | --- |
| 1 | | | 0.30 | 0.25 |
| 2 | | | 0.17 | 0.10 |
| 3 | | | 0.25 | 0.20 |
| 4 | | | 0.17 | 0.08 |
| 5 | | | 0.19 | 0.13 |
| 6 | | | 0.27 | 0.20 |
| 7 | | | 0.13 | 0.07 |
| 8 | | | 0.07 | 0.00 |
| 9 | | | 0.40 | 0.27 |
| 10 | | | 0.27 | 0.20 |
| 11 | | | 0.32 | 0.27 |
| 12 | | | 0.67 | 0.44 |
| 13 | | | 0.35 | 0.30 |
| 14 | | | 0.35 | 0.32 |
| 15 | | | 0.07 | 0.00 |
| 16 | | | 1.30 | 0.84 |
| 17 | | | 0.15 | 0.07 |
| 18 | | | 0.86 | 0.78 |
| 19 | | | 0.73 | 0.67 |
| 20 | | | 0.66 | 0.61 |
| 21 | | | 0.56 | 0.47 |
| 22 | | | 0.52 | 0.47 |
| 23 | | | 0.84 | 0.64 |
| 24 | | | 0.91 | 0.84 |
| 25 | | | 0.62 | 0.61 |
| 26 | | | 0.25 | 0.19 |
| 27 | | | 0.24 | 0.20 |
| 28 | | | 0.32 | 0.22 |
| 29 | | | 0.57 | 0.51 |
| 30 | | | 0.13 | 0.00 |
| **Median [IQ]** | | | **0.32[0.18-0.62]** | **0.26[0.13-0.50]** |
|  |  |  | | |

Baseline_High_: baseline at the higher dose of norepinephrine, Baseline_Low_: baseline at the lower dose of norepinephrine, IQ: interquartile, NE: norepinephrine.

Supplementary figure 1. The heart-Lung interactions method for estimating the determinants of venous return

Example of the estimation of the venous return curve and its determinants with the heart-lung interactions method in a typical patient.

CI: cardiac index, CVP: central venous pressure, Pms: mean systemic pressure, RAP: right atrial pressure.

Supplementary figure 2: Correlation between the changes in mean systemic pressure induced by decreasing the dose of norepinephrine and the amplitude of the dose decrease

N=30. Pms: mean systemic pressure, NE: norepinephrine
